# The Influence of Emotion in the Management of Amateur Football Organizations

**DOI:** 10.3389/fpsyg.2020.02218

**Published:** 2020-09-04

**Authors:** Melany Hebles, Vicente Javier Prado-Gascó, Orlando Llanos-Contreras, Mario Alguacil

**Affiliations:** ^1^Management Department, Faculty of Economics and Management Science, Catholic University of the Most Holy Conception, Concepción, Chile; ^2^Social Psychology Department, Faculty of Psychology, University of Valencia, Valencia, Spain; ^3^Department of Teaching and Learning of Physical, Plastic and Musical Education, Faculty of Teaching and Educational Sciences, Catholic University of Valencia San Vicente Mártir, Valencia, Spain

**Keywords:** affective commitment, emotional attachment, emotions in sport management, leadership, loyalty, organizational identification, service quality, sport management

## Abstract

This article is oriented to the analysis of organizational and emotional variables in amateur sporting organizations. The general objective is to analyze the influence of organizational variables such as service quality, transactional leadership, and transformational leadership and emotional variables such as affective commitment, emotional attachment investment, and emotional attachment dividend to predict the credibility that members of amateur sporting organizations perceive, as well as their degree of identification and loyalty. The opinions of 203 members of Chilean amateur football teams [169 men and 34 women, with ages between 18 and 68 years (mean = 32.75 years, DT = 9.92)] have been analyzed through a self-completed questionnaire. To reach the objectives, two types of differential but complementary analyses, in the form of hierarchical regression models (from hereon, HRMs) and qualitative comparative analysis (from hereon, QCA), were performed. The results obtained suggest that the organizational variables are better predictors than the emotional variables in all of the cases. In the same way, the inclusion of the emotional variables improves the predictive capacity of the proposed models to explain identification and loyalty, but not in the case of credibility. In general, the variables considered seem to explain 37% of the credibility, 56% of loyalty, and 65% of identification. On the other hand, considering the results of the QCA, no variable turned out to be necessary. However, different combinations of variables (conditions) were observed that were able to explain between 47 and 91% of the cases of the variables analyzed. In general, based on these results, it was observed that the emotional variables were important in interaction with other organizational ones since they are present in the three combinations that most explain identification and loyalty and are also present in the three combinations that most explain credibility. This study contributes to the literature by supporting the importance of managing emotions in order for sporting organizations to be more successful.

## Introduction

Emotions can be a source of conflict within an organization, as they can also be an element capable of promoting the generation of social capital, trust among members of the same team, and a common purpose to follow (Llanos-Contreras and Jabri, 2019; Chang, 2020). Emotions are the result of the formation, interruption, or renovation of affective links and are considered a central element that allows for reinforcing the connections between people (Grisaffe and Nguyen, 2011). Therefore, the managing of emotions would be critical in strengthening the effects of organizational management in the performance of an organization or team. In this way, emotions are a basic driver that must be kept in mind in contemporary society, as well as in sporting organizations. People are connected, and for this reason, the decisions they make affect others in the organization, influencing their own emotions and actions related to behavioral decisions such as credibility, loyalty, and identification (Rodríguez-Pomeda et al., 2017).

For their part, credibility, loyalty, and identification of the members of an organization inform about the behaviors associated with the commitment of the people with an organization and influence the stability and success of the teams (Punjaisri and Wilson, 2011; Del Barrio-García and Prados-Peña, 2019). Credibility increases the levels of trust of the stakeholders and is fundamental in sustaining lasting personal relationships (Connelly et al., 2011). Loyalty is related to the psychological commitment of the members of an organization (Jacoby, 1971), and the identification with the organization is associated with high levels of positive feelings such as belonging (Punjaisri and Wilson, 2011). A better position concerning these three indicators is undoubtedly desirable, and understanding which aspects must be managed is central to achieving it.

The literature about management of emotions in sporting entities has advanced in understanding these elements from a commercial perspective in organizations with high levels of professionalization (e.g., Rodríguez-Pomeda et al., 2017). The importance of emotions in the willingness to align oneself with a determined club has been researched as has the identification of the fans with their club (Dwyer et al., 2015). The literature has also advanced in the comprehension of aspects of organizational management and leadership in the performance of elite sports (Arnold et al., 2015). However, little is known about the influence of organizational and emotional aspects in sporting organizations when these are not professional.

Among the organizational factors that influence the behavior and commitment of the people with an organization or team are found styles of transformational leadership and transactional leadership (Peng et al., 2020). Another organizational variable that is relevant for this case is the service quality, and among the emotional variables, there are affective commitment, emotional attachment investment, and emotional attachment dividend (Calabuig Moreno et al., 2008; Dwyer et al., 2015). For their impact in the sporting and social success of these entities, it is important to understand how these organizational and emotional variables influence credibility, identification, and loyalty, as well as their relative importance for this effect. In order to advance in the comprehension of this phenomenon, this study seeks to respond to the questions of how organizational variables (service quality, transactional leadership, and transformational leadership) and emotional variables (affective commitment, emotional attachment investment, and emotional attachment dividend) influence credibility, identification, and loyalty in members of amateur sporting organizations and which of them are more important in explaining the variables of interest.

To respond to this question, three prediction models were tested in two steps. In the first step, considering the organizational variables (service quality, transactional, and transformational leadership) and, in the second, adding the emotional variables. This allowed us to test, on the one hand, if the organizational variables significantly predict the variables of interest and, on the other hand, if the inclusion of the emotional variables improves the predictive capability of the models significantly. Besides, a comparative qualitative analysis was developed to know if there exists a necessary variable which must always be present to produce the expected result and which variables, or interactions of variables, are sufficient to reach the expected result.

This article is developed in this way; what follows is a theoretical discussion about the organizational and emotional variables being studied and their potential impact on the variables of interest, then we inform about the methodological aspects of the study with the following section showing the results obtained from the analysis of the data. The last section discusses the results and presents the main conclusions.

### Theoretical Framework

The perceived service quality is a factor that has been used to explain behavior and organizational performance at a business level and also in the context of sporting organizations, as well as in events (Calabuig et al., 2014) and services (García-Fernández et al., 2018). The perceived service quality has to do with the fulfillment of expectations and the level of satisfaction of users-members of sporting entities (Grönroos, 1984). Both the way the club offers services and the functioning of the organization on an internal level (accesses to the stadium, parking, cleaning, quality of the playing field, repair services, and fan control) must be taken into account, as both aspects contribute to the satisfaction of the fans (Nogales, 2006). These are the attributes used over and over again by users-members to refer to the quality of the service and their satisfaction with it (Kelly and Turley, 2001).

There is literature that relates the perceived service quality (Castillo-Rodríguez et al., 2019) with different organizational variables such as value (Oriade and Schofield, 2019) and satisfaction with a sporting organization (Theodorakis et al., 2019; Vuong et al., 2020), as well as the relationships between them and the future intentions of the users (Crespo-Hervás et al., 2019) which are essential for the success of an event and/or service in the sporting sphere. Along the same lines, it would seem that the service quality influences loyalty and the level of participation of the members of sporting clubs (Alexandris et al., 2017), as well as in the perceived credibility of the entity (Alguacil et al., 2018). In the same way, the existing literature suggests the existence of a positive relationship between the perception of the service quality of a sporting organization and the identification of the spectators (Benesbordi and Esmaeili, 2019).

In summary, the research analyzed above points out the importance of the service quality in relation to sporting entities and their users. This research confirms the existence of a positive relationship between the service quality and other organizational results associated with the participants. These relationships suggest the following hypothesis.

H1: The service quality positively and significantly influences credibility, loyalty, and identification.

The literature on styles of leadership and organizational management informs of the existence of relationships between leadership, management, and business results (Fletcher and Arnold, 2015). When talking of leadership, following the classic definitions, this concept is understood as a behavioral process in which the leader seeks influence over individuals in order to obtain predetermined objectives (Barrow, 1977), whether these be at an organizational or social level (Hollander, 1985). The literature offers multiple classifications of leadership styles, and among the most used is that which argues that a leader can be more oriented either to the task or to the people (Blake and Mouton, 1964). This defines leadership in terms of the types of interactions carried out by the leader with members of his or her team, deriving in two categories of leadership, transactional leadership and transformational leadership (Avolio et al., 1999).

Transactional leadership has to do with a strong style of management, based on reward and punishment, where the rules to comply to are clearly established and where the relationships between members of the group are based on extrinsic aspects, such as the economic aspect (Si and Wei, 2012). In transformational leadership, the leader is charismatic and more ambitious in the proposed objectives, where persuasion is exercised to convince the group that those objectives are reachable (Bernerth and Hirschfeld, 2016). These styles of leadership are widely studied for their influence in worker performance and organizational behavior (Lee and Ding, 2020). In sport and the management of sporting organizations, the styles of leadership are especially relevant as they affect the motivations of the sportspeople and the relationships they establish with their leaders, which in turn boost the success of some teams over others (Arnold et al., 2015; Mitrovic et al., 2019). Therefore, the understanding of how these styles of leadership affect organizational results helps to satisfy the needs of the sporting organization and also to overcome challenges that surround not-for-profit sports such as organizational commitment (Peng et al., 2020).

Transactional leadership has been traditionally considered as an antagonist of transformational leadership, granting it a negative connotation in most cases (Laohavichien et al., 2009). However, there exists evidence that shows that both leadership styles can be useful and complementary in improving management and organizational performance (e.g., Hetland and Sandal, 2003; Xu and Wang, 2019). Thus, even when transactional leadership is useful mainly to improve the response to individuals in terms of a task (Lee and Ding, 2020), this would also positively influence variables associated with behavior and the commitment of individuals such as motivation and loyalty. Along these lines, Monzani et al. (2014) have found that while transformational leadership is a better precursor to loyalty, there are no significant differences between them when the followers present a high level of affability, kindness, and respect. In the same way, Epitropaki and Martin (2005) found a positive relationship between transactional leadership and organizational identification, particularly for individuals characterized by a connected scheme of self.

According to Lee and Ding (2020), there is little information that measures the relationship between specific styles of leadership and results that are reflected in people’s behavior. However, the literature offers empirical evidence that would support this idea. Mitrovic et al. (2019) inform that the leadership style and the improvement in the relationships between the sporting management and the members of the organization positively affect motivation, mutual trust, and loyalty of the members. Particularly, in terms of transformational leadership, Bass and Riggio (2006) and Caillier and Sa (2017) have demonstrated that there exists a positive impact from this style of leadership on motivation, attitudes, and behavior of employs, as well as in commitment. In the same way, Peng et al. (2020) found that transformational leadership has a direct bearing on how employs perceive their work and their emotional commitment to the organization. Other empirical studies have demonstrated that the transformational style of leadership is positively associated with the satisfaction of followers with their leaders and with the work in itself (Lee et al., 2018), as well as with the credibility perceived by the members and the behaviors they carry out (Bolkan and Goodboy, 2009).

The aforementioned shows that transformational leadership and transactional leadership positively influence variables that reflect different behaviors that are associated with commitment and loyalty to an organization, which suggests the following hypothesis:

H2: Transformational leadership and transactional leadership positively and significantly influence credibility, loyalty, and identification.

The *affective commitment* of people is a central element in defining attitudes and social behavior of individuals (Agrawal and Maheswaran, 2005). A greater or lesser level of affective commitment is related to positive or negative attitudes in terms of a person, organization, brand, company, or institution. Affective commitment is defined as “a psychological state internal to the individual, which is the result of an initial attraction process” (Heere and Dickson, 2008, p. 228). This has to do with the intention of individuals to establish lasting relationships with others (Morgan and Hunt, 1994). It is also related to those emotions that are linked with the relationship already established with a brand, organization, service, team, or other (Geyskens et al., 1996).

Affective commitment has been demonstrated to be a relevant predictor in the performance of volunteer workers in non-profit organizations (Hoye, 2007). This would occur because the affective commitment of the volunteers generates greater stability in their link with the organization (Preston and Brown, 2004). In the case of amateur clubs, this factor would be central in recruiting and adequate management of volunteers, as well as in their capacity to generate activities that allow its viability. Affective commitment is considered a central element to explain the loyalty and satisfaction of the individuals that participate in non-profit organizations (Juaneda-Ayensa et al., 2017). Along the same lines, affective commitment is associated with the motivation of the members of these organizations in the development of their volunteer activities (McCormick and Donohue, 2019). Therefore, agreement exists in the literature on the importance of this factor in the behavioural decisions of the individuals.

The systematic nature of people’s behavior means that emotions are a central factor in explaining the decisions and social behavior of the individual. How some members of an organization feel and behave will have an influence on the actions and emotions of others in the team (Rodríguez-Pomeda et al., 2017). Particularly, emotional attachment is an important factor in explaining the connections between people, human relationships, and the emotional consequences of these relationships (Bowlby, 1982; Shaver and Mikulincer, 2002). If this emotional attachment does exist, there could be a commitment that leads to greater willingness of people to make personal sacrifices to achieve objectives within an organization (Chatzopoulou and Tsogas, 2017). In the same way, high levels of emotional attachment will generate high levels of identification with a group or product (Paxton and Moody, 2003) and greater levels of perceived credibility (Dwivedi et al., 2019).

Emotional attachment is composed of two distinct forms of attachment. One, known as emotional attachment investment, is considered as the cognitive feelings put into the team and the other, known as emotional attachment dividend, is understood as the affective feelings derived from the team (Dwyer et al., 2015). Understanding the differences in emotional attachment, whether it is affective or cognitive, offers relevant information on the behavior of the fans as these emotions influence the mood of the people and predict their loyalty to a sporting institution (Fedorikhin et al., 2008). This is considered a critical factor for the management of this type of institution.

In the sphere of football, like in other sports, there are strong emotional responses where the spectators create a link with the entity, receiving in return intangible elements in the form of emotions (Hay and McDonald, 2007). For this reason, to the extent that the fans and participants are linked, their levels of satisfaction increase, and their emotional attachment grows, positively influencing their levels of loyalty (Dwyer et al., 2015). This same thing could occur in the case of sportspeople linked to amateur institutions, as they also compete defending their specific club many times also as fans. This discussion leads to the following hypothesis.

H3: Affective commitment and emotional attachment (investment and dividend) positively and significantly affect credibility, loyalty, and identification

Understanding the management of emotion in sporting organizations is a challenging topic as it is believed to be an important element for the role that fans’ and sportspeople’s emotions, such as attraction and passion, play in the results of the organization (Rodríguez-Pomeda et al., 2017). According to Zajonc and Markus (1982), people develop preferences based on what they think and feel with respect to an organization, brand, or service. While the importance of the cognitive aspects and the management in the definition of these preferences are recognized, these aspects fade over time while the emotional factors tend to last (Park and MacInnis, 2006). Particularly, for the case of sporting organizations, when the fact is added that the strength of the emotions has an individual effect in the participants and fans of the football clubs, but also generates a collective effect of identification with the club. This would have an influence on what happens on the inside of the institution (Fink et al., 2002). Thus, distinct to the management variables whose effect is considered more linear, the emotional variables would generate multiple effects that are reinforced at an individual and collective level. Therefore, affective commitment and emotional attachment would be a better predictor of credibility, loyalty, and identification, leading to the following hypothesis.

H4: The emotional variables of emotional attachment (investment and dividend) and affective commitment are better predictors of credibility, loyalty, and identification than the management variables (service quality, transformational leadership, and transactional leadership).

## Materials and Methods

### Participants

The sample for this study consisted of 203 members of Chilean football clubs, ranging in age from 18 to 68, with an average age of 32.75 (±9.92), 83% (169) of whom were men. On the other hand, in terms of the labor, 73.4% (*n* = 149) were in full-time employment, 19.2% were part-time workers, 4.4% were unemployed, 2% were students, and 1% were retirees/pensioners.

On the other hand, with regard to the series of the club in which they participate, 19.2% (*n* = 39) belonged to Honor series, 16.7% corresponded to women’s football, 11.3% were from second and first age adult series, 10.8% belonged to Super Senior, while at third age adult level, there were 16 (7.9%), 4.9% were from Senior level, and from the Golden Age, there was only one person who represents 0.5% of the total.

Finally, with regard to the participants’ involvement in the club (Table 1), the participants belonged to the club between 0 and 54 years, with an average of 10.12 years (±11.55). On the other hand, in relation to the monthly frequency of playing in the club, those surveyed play 3.91 days mean (±1.30) from those who only attend 1 day a month to those who attend up to 10 occasions. Finally, in terms of the frequency with which they participate in the activities organized by the club, apart from matches, we can see that the mean participation is 2.68 times a month (±1.80) from those who participate once a month to those who participate eight times.

**TABLE 1:** Description in relation to belonging and frequency of playing and participation in activities of the club.

	Belonging to the club (years)	Playing in the club (month)	Participation club activities (month)
Mean	10.12	3.91	2.68
SD	11.55	1.30	1.80
Minimum	0	1	1
Maximum	54	10	8

### Instrument

Data for this study were collected between September 2019 and February 2020. The instrument used in the study is composed of a series of questionnaires with a scale of Likert-type responses of five anchors, where 1 means totally disagree with the statement and 5 totally agree. The filling time of the instrument was 40 min. All the scales have shown adequate psychometrics both in the aforementioned contributions, meeting the criteria of reliability, meeting the composed reliability, Cronbach’s alpha (Hair et al., 2006) and the AVE value (Fornell and Larcker, 1981), as well as in the criteria of validity, such as the root mean square error of approximation (RMSEA) value (Browne and Cudeck, 1993), the fit indexes (Hu and Bentler, 1999), the weighting of items in each Scale (Bagozzi et al., 1998), and the T values (Veasna et al., 2013).

*Scale of service quality* is made up of five items taken from Yoo and Donthu (2001), Carrasco and Gutiérrez (2008), and Lee and Leh (2011). Scores range from 1 to 5, with higher scores indicating a higher perceived service quality. The Scale has shown adequate psychometric properties in previous studies (Lee and Leh, 2011), which are also observed in this study (α = 0.83).

*Scale of affective commitment*, adapted from Tuškej et al. (2013), is made up of three items, with scores ranging from 1 to 5. Higher scores indicate a greater affective commitment with the club. The Scale has shown adequate psychometric properties in previous studies (Tuškej et al., 2013), which are also shown in the present one (α = 0.83).

*Scale of emotional attachment*, adapted by the research team from Dwyer et al. (2015), is composed of seven items grouped into two factors: emotional attachment investment (three items) and emotional attachment dividend (four items). Scores range from 1 to 5, with higher scores indicating a greater emotional attachment with the club. The Scale has shown adequate psychometric properties both in previous studies (Dwyer et al., 2015) and in the present one (emotional attachment investment, α = 0.86; emotional attachment dividend, α = 0.91).

*Scales of leadership* are adapted from the work of Avolio and Bass (2004). The scale scores of transformational leadership (seven items) and that of transactional leadership (six items) range from 1 to 5, with higher scores indicating a greater transformational or transactional leadership perceived in the club. The scales have shown adequate psychometric properties both in previous studies (Avolio and Bass, 2004) as well as in the present one (transformational leadership, α = 0.94; transactional leadership, α = 0.92).

*Scale of brand credibility* is composed of six items adapted by the research team from Sweeney and Swait (2008). Scores range from 1 to 5, with higher scores indicating greater credibility in the club. The Scale has shown adequate psychometric properties in previous studies (Sweeney and Swait, 2008), something that is also seen in the present study (α = 0.82).

*Scale of brand loyalty* has five items adapted by the research team taken from Yoo and Donthu (2001); Tong and Hawley (2009), and Lee and Leh (2011). Scores range from 1 to 5, with higher scores indicating greater loyalty toward the brand or service. The Scale has shown adequate psychometric properties in previous studies (Yoo and Donthu, 2001; Tong and Hawley, 2009; Lee and Leh, 2011), as well as in this study (α = 0.82).

*Scale of brand identification* is composed of five items taken from Stokburger-Sauer et al. (2012). Scores range from 1 to 5, with higher scores indicating a greater identification with the club. The Scale has shown adequate psychometric properties both in previous studies (Stokburger-Sauer et al., 2012) and in the present one (α = 0.89).

### Statistical Analysis

Firstly, three models of hierarchical regression were carried out in two steps (credibility, loyalty, and identification). In the first step, the organizational variables were included (service quality, transformational leadership, and transactional leadership). In the second, the emotional variables (affective commitment, emotional attachment investment, and emotional attachment dividend) were included. Then a qualitative comparative analysis (QCA) was carried out. For this purpose, the data collected from the responses of the participants were transformed into fuzzy set responses. For this, the first task was to eliminate all the lost data (five participants) to then calculate the different constructs by multiplying the items that make them up (Giménez-Espert and Prado-Gascó, 2018). Once done, the values were recalibrated through the fsQCA 3.0 software (Ragin and Davey, 2016), calibrating said values between 0 and 1 (Ragin, 2008) following the consideration of Rey-Martí et al. (2016) and Woodside (2013) that argue for the need to establish three thresholds: 10% (low level of agreement or totally outside the set), 50% (degree of intermediate agreement, neither inside nor outside the set), and 90% (high level of agreement, completely inside the set). After the recalibration of the values of the variables, we proceeded to carry out the analysis of need and sufficiency. In the analysis of need, the intention is to test if any of the variables that form part of the study must be present in all of the cases to obtain the expected result, understanding that a variable is necessary when the consistency values of that variable are above the criterion of 0.90 (Ragin, 2008). For its part, the analysis of sufficiency aims to show those conditions or interactions of conditions that lead to a specific result. For the calculation of said sufficient conditions, Eng and Woodside (2012) indicate that two steps should be produced: the first of these is to generate a table of the truth in which the scores of the diffuse sets are transformed into the combinations of causal conditions that are logically possible and their empirical result. In the second step, three solutions are generated: complex (the most restrictive), parsimonious (the least restrictive), and intermediate, which is the one recommended in the literature (Ragin, 2008). In this analysis of sufficiency, paths or combinations are shown that explain the variance of the variables of interest, finding three fundamental terms: the consistency of the solution, which has to do with the possible reliability or fit of the model, and must be above 0.75 to consider the solution adequate. On the other hand, there is the raw coverage that indicates how many cases can be explained by each combination of conditions, while the unique coverage is the variance that can be explained by a concrete combination of conditions and not by other possible combinations (Eng and Woodside, 2012). The software used has been, on the one hand, the statistical package SPSS (Statistical Package for the Social Sciences, Version 25, Armonk, NY, United States: IBM Corp.) for the descriptive analysis, the HRM models, and the obtaining of the calibration values, and, on the other hand, the fsQCA (Fuzzy-Set Qualitative Comparative Analysis, version 3.0, Irvine, CA, United States; Ragin and Davey, 2016, Department of Sociology, University of California) for the qualitative comparative analysis.

### Ethical Considerations

This study respected the fundamental principles of the Declaration of Helsinki (World Medical Association [WMA], 2013), with particular emphasis on the anonymization of the data collected, confidentiality, and non-discrimination of participants. The protocol was approved by the Scientific Ethics Committee of the Catholic University of the Holy Conception, Chile.

## Results

In order to achieve the objectives of the study and to test the four hypotheses raised, two differential analyses, hierarchical regression models and comparative qualitative models, were carried out.

### Hierarchical Regression Models

Based on the results obtained from the HRM (Table 2), it is observed how the prediction of the considered models oscillates between 37 and 65%. The variables taken into account are better predictors in the case of brand identification (*R*^2^_*adjusted*_ = 0.65, *p* = 0.000), followed by brand loyalty (*R*^2^_*adjusted*_ = 0.56, *p* = 0.000), with the case of brand credibility being where they predicted least (*R*^2^_*adjusted*_ = 0.37, *p* = 0.000). In general, the inclusion of the emotional variables improved the prediction of brand loyalty and brand identification, but without producing a significant improvement in the model in the case of brand credibility.

**TABLE 2:** Hierarchical regression models for the prediction of brand credibility, brand loyalty, and brand identification.

Variable	Brand credibility		Brand loyalty		Brand identification	
Predictors	ΔR^2^	β	ΔR^2^	β	ΔR^2^	B
Step 1	0.39***		0.37***		0.39***	
SQ		0.41***		0.22**		0.24**
TFL		0.23**		0.44***		0.43***
TSL		0.09		0.02		0.03
Step 2	0.007		0.20***		0.26***	
SQ		0.38***		–0.03		–0.06
TFL		0.18*		0.01		0.02
TSL		0.09		0.03		0.03
AC		0.14		0.15		0.44***
EAI		–0.04		0.30***		0.23**
EAD		–0.01		0.31***		0.24***
Total R^2^_*adjusted*_	0.37		0.56***		0.65***	

*p < 0.05; **p < 0.01; ***p < 0.001. “−,” not part of the analysis; SQ, service quality; TFL, transformational leadership; TSL, transactional leadership; AC, affective commitment; EAI, emotional attachment investment; EAD, emotional attachment dividend.

In concrete terms, in the first of the predictions, that of brand credibility, we see that, in the first step, the model is capable of predicting 39% of the variance of the brand credibility (*R*^2^_*adjusted*_ = 0.39) where the variable of service quality exercises the greatest weight (β = 0.41; *p* = 0.000) followed by transformational leadership (β = 0.23; *p* = 0.003). In the second step, the inclusion of the emotional variables does not improve the model (ΔR^2^ = 0.007, *p* = 0.533), and the final model is able to explain 37% of the variance of brand credibility (*R*^2^_*adjusted*_ = 0.37), with service quality (β = 0.38; *p* = 0.000) and transformational leadership (β = 0.18; *p* = 0.042) as influential.

In the second hierarchical regression, that of the prediction of brand loyalty, we see that the initial variables are capable of predicting 37% of the variance of brand loyalty (*R*^2^_*adjusted*_ = 0.37) where a significant influence is shown by the variables of transformational leadership (β = 0.44; *p* = 0.000) and service quality (β = 0.22; *p* = 0.003). With the inclusion of the emotional variables in the second step, the model improves (ΔR^2^ = 0.20, *p* = 0.001), and thus the model is finally capable of predicting 56% of the variance of brand loyalty (*R*^2^_*adjusted*_ = 0.56). The variables that significantly influence the prediction are the emotional attachment dividend (β = 0.31; *p* = 0.000) and the emotional attachment investment (β = 0.30; *p* = 0.000).

Lastly, we find the prediction model for brand identification in which the first step of the analysis indicates that the model predicts 39% of the variance of that variable (*R*^2^_*adjusted*_ = 0.39) where transformational leadership (β = 0.43; *p* = 0.000) and service quality (β = 0.24; *p* = 0.001) significantly influence prediction. In the second step, again including the emotional variables, we see that the model improves (ΔR^2^ = 0.26, *p* = 0.000) and is able to predict on this occasion up to 65% of the variance of that variable (*R*^2^_*adjusted*_ = 0.65), where affective commitment (β = 0.44; *p* = 0.000) shows the highest weight, followed by emotional attachment dividend (β = 0.24; *p* = 0.000) and emotional attachment investment (β = 0.23; *p* = 0.001).

### Fuzzy-Set Qualitative Comparative Analysis

To carry out the qualitative comparative analysis, as has already been set out, firstly, the descriptors and calibration values were calculated for the variables under study (Table 3). Then we proceeded to carry out the analysis of need and sufficiency.

**TABLE 3:** Descriptive statistics and calibration values.

		BC	BL	BI	AC	SQ	EAI	EAD	TFL	TSL
Mean		7,845.63	2,053.86	1,982.83	90.62	1,687.71	91.19	459.35	26,345.07	115,298.11
SD		4,927.16	984.21	1,006.32	35.03	919.94	35.61	190.84	16,299.10	185,626.36
Minimum		1	1	1	1	1	1	1	1	1
Maximum		15,625	3,125	3,125	125	3,125	125	625	46,875	2,156,250
*Calibration values*										
Percentiles	10	1,728	625	518.4	39.6	432	36	144	3,142.8	8,326.8
	50	8,000	2,000	2,000	100	1,600	100	500	24,000	82,048
	90	15,625	3,125	3,125	125	3,125	125	625	46,875	203,125

*BC, brand credibility; BL, brand loyalty; BI, brand identification; SQ, service quality; TFL, transformational leadership; TSL, transactional leadership; AC, affective commitment; EAI, emotional attachment investment; EAD, emotional attachment dividend.*

#### Analysis of Necessity for Brand Credibility, Brand Loyalty, and Brand Identification

Based on the results obtained (Table 4), none of the variables or conditions turned out to be necessary to obtain high levels (presence) or low levels (absence) of credibility, loyalty, and identification, as the consistency in all of the cases was less than 0.90 (Ragin, 2008).

**TABLE 4:** Analysis of necessity for brand credibility, brand loyalty, and brand identification.

	BC		∼BC		BL		∼BL		BI		∼BI	
	Con	Cov	Con	Cov	Con	Cov	Con	Cov	Con	Cov	Con	Cov
SQ	0.80	0.74	0.49	0.52	0.71	0.78	0.49	0.45	0.72	0.76	0.50	0.47
∼SQ	0.49	0.45	0.76	0.81	0.50	0.54	0.75	0.68	0.50	0.53	0.75	0.71
TFL	0.79	0.70	0.51	0.52	0.77	0.80	0.46	0.40	0.79	0.80	0.47	0.42
∼TFL	0.45	0.45	0.70	0.79	0.42	0.49	0.77	0.74	0.42	0.47	0.77	0.77
TSL	0.78	0.72	0.48	0.51	0.71	0.78	0.47	0.42	0.74	0.79	0.45	0.43
∼TSL	0.47	0.44	0.74	0.79	0.48	0.52	0.76	0.69	0.46	0.49	0.77	0.73
AC	0.77	0.69	0.49	0.50	0.78	0.82	0.41	0.36	0.83	0.84	0.39	0.36
∼AC	0.45	0.43	0.70	0.78	0.39	0.45	0.80	0.75	0.37	0.40	0.83	0.81
EAI	0.75	0.65	0.54	0.54	0.82	0.84	0.42	0.35	0.82	0.81	0.43	0.38
∼EAI	0.47	0.47	0.65	0.75	0.37	0.43	0.81	0.78	0.37	0.42	0.78	0.79
EAD	0.80	0.65	0.56	0.53	0.87	0.83	0.44	0.35	0.87	0.81	0.45	0.37
∼EAD	0.42	0.46	0.63	0.79	0.32	0.42	0.79	0.83	0.32	0.39	0.76	0.84

*∼, absence of condition; Con, consistency; Cov, coverage; Condition needed, consistency ≥0.90; BC, brand credibility; BL, brand loyalty; BI, brand identification; SQ, service quality; TFL, transformational leadership; TSL, transactional leadership; AC, affective commitment; EAI, emotional attachment investment; EAD, emotional attachment dividend.*

#### Analysis of Sufficiency of Credibility, Loyalty, and Identification

Once the analysis of necessity had been carried out, we proceeded to the analysis of sufficiency. As can be seen in Table 5, the frequency cutoff was set at 1, and the cutoffs for consistency are found in the range between the value of 0.82 and the value of 0.90. In the same table, the three most important combinations for the presence and absence of each of the conditions (brand credibility, brand loyalty, and brand identification) are summarized.

**TABLE 5:** Analysis of sufficiency for credibility, loyalty, and identification.

*Frequency cutoff: 1*	BC			∼BC			BL			∼BL			BI			∼BI		
	*Consistency* *cutoff: 0.87*			*Consistency* *cutoff: 0.89*			*Consistency* *cutoff: 0.83*			*Consistency* *cutoff: 0.90*			*Consistency* *cutoff: 0.82*			*Consistency* *cutoff: 0.90*		
	1	2	3	1	2	3	1	2	3	1	2	3	1	2	3	1	2	3
SQ	•	•	−	∘	∘	−	−	•	−	−	∘	∘	−	−	−	−	−	−
TFL	•	∘	•	−	−	−	•	−	−	−	−	−	•	−	−	−	−	∘
TSL	∘	•	∘	∘	−	∘	−	−	•	−	−	∘	−	−	•	−	−	•
AC	−	−	−	−	−	−	−	−	−	−	∘	−	−	•	−	∘	−	−
EAI	−	−	−	−	∘	−	−	•	−	−	−	∘	−	•	−	−	−	∘
EAD	−	−	•	−	−	∘	•	•	•	∘	−	−	•	•	•	−	∘	−
Consistency	0.83	0.89	0.80	0.88	0.87	0.86	0.87	0.91	0.86	0.83	0.81	0.87	0.86	0.92	0.85	0.81	0.84	0.89
Raw coverage	0.30	0.29	0.29	0.64	0.59	0.58	0.72	0.67	0.67	0.79	0.67	0.61	0.73	0.70	0.69	0.83	0.76	0.27
Unique coverage	0.01	0.03	0.002	0.03	0.02	0.02	0.01	0.03	0.02	0.13	0.01	0.004	0.01	0.03	0.01	0.12	0.05	0.007
Overall solution consistency	0.75			0.79			0.83			0.75			0.81					0.77
Overall solution coverage	0.47			0.77			0.86			0.85			0.88					0.91

*•, presence of condition; ∘, absence of condition. *All sufficient conditions are adequate, raw coverage between 0.47 and 0.91.*∼, low levels of condition; BC, brand credibility; BL, brand loyalty; BI, brand identification; SQ, service quality; TFL, transformational leadership; TSL, transactional leadership; AC, affective commitment; EAI, emotional attachment investment; EAD, emotional attachment dividend. Expected vector for credibility: 1.1.1.1.1 (0, absent; 1, present). Expected vector for ∼ credibility: 0.0.0.0.0. Expected vector for loyalty: 1.1.1.1 (0, absent; 1, present). Expected vector for ∼ loyalty: 0.0.0.0. Expected vector for identification: 1.1.1.1 (0, absent; 1, present). Expected vector for ∼ identification: 0.0.0.0. Using the format of Fiss (2011).*

Firstly, in terms of the prediction of high levels of credibility, we found seven paths or interactions capable of explaining 47% of the cases, where the three most representative combinations were: (a) the interaction between high service quality, high levels of transformational leadership, and low levels of transactional leadership that explained 30% of the cases (Raw coverage = 0.30; Consistency = 0.83); (b) the interaction between high levels of service quality, low levels of transformational leadership, and high levels of transactional leadership that explained 29% of the cases (Raw coverage = 0.29; Consistency = 0.89); and (c) the interaction between high levels of transformational leadership, low levels of transactional leadership, and high levels of emotional attachment dividend that explained 29% of the cases (Raw coverage = 0.29; Consistency = 0.80). On the other hand, in terms of the prediction of low levels of brand credibility, of the six paths capable of explaining 77%, the three most representative interactions were: (a) low levels of service quality and low levels of transactional leadership (Raw coverage = 0.64; Consistency = 0.88), (b) low levels of service quality and low levels of emotional attachment investment (Raw coverage = 0.59; Consistency = 0.87), and (c) low levels of transactional leadership and low levels of emotional attachment dividend (Raw coverage = 0.58; Consistency = 0.86) that explained 64, 59, and 58% of the cases, respectively.

Secondly, in terms of the prediction of high levels of brand loyalty, seven combinations explained 86% of the cases, with the three most important interactions being: (a) the interaction between high levels of transformational leadership and high levels of emotional attachment dividend (Raw coverage = 0.72; Consistency = 0.87), (b) high levels of service quality with high levels of emotional attachment, both investment and dividend (Raw coverage = 0.67; Consistency = 0.91), and (c) high levels of transactional leadership and high levels of emotional attachment dividend (Raw coverage = 0.67; Consistency = 0.86), of which 72% of the cases were explained by the first interaction and 67% by each of the remaining. In terms of the prediction of low levels of loyalty, four paths were obtained that could explain 85% of the cases, with the three most relevant interaction being: (a) low levels of emotional attachment dividend (Raw coverage = 0.79; Consistency = 0.83) that explained 79% of the cases, (b) the interaction of low levels of service quality and commitment (Raw coverage = 0.67; Consistency = 0.81) that explained 67% of the cases, and, finally, (c) the interaction between low levels of service quality together with low levels of transactional leadership and low levels of emotional attachment investment (Raw coverage = 0.61; Consistency = 0.87) that explained 61% of the cases.

Finally, for the prediction of high levels of brand identification with the club, seven paths were found that could explain 88% of the cases. Among them, the three most relevant interactions were: (a) the interaction of high levels of transformational leadership and high levels of emotional attachment dividend (Raw coverage = 0.73; Consistency = 0.86) that explained 73% of the cases; (b) the interaction of high levels of commitment with high levels of attachment, both investment and dividend (Raw coverage = 0.70; Consistency = 0.92) that explained 70% of the cases; and, finally, (c) the interaction between high levels of transactional leadership with high levels of emotional attachment dividend (Raw coverage = 0.69; Consistency = 0.85) that explained 69% of the cases. In terms of the prediction of low levels of identification with the club, three paths were found that explained 91% of the cases. These paths were: (a) low levels of commitment (Raw coverage = 0.83; Consistency = 0.81) that explained 83% of the cases; (b) low levels of emotional attachment dividend (Raw coverage = 0.76; Consistency = 0.84) that explained 76% of the cases; and, finally, (c) the interaction between low levels of transformational leadership, high levels of transactional leadership, and low levels of emotional attachment investment (Raw coverage = 0.27; Consistency = 0.89) that explained 27% of the cases.

## Discussion and Conclusion

The aim of this work has been two-pronged; on the one hand, it sought to determine how organizational and emotional variables influence the credibility, loyalty, and identification of members of amateur sporting organizations and, on the other hand, it has wanted to determine which of them were more important in explaining the variables of interest. The analyses carried out by means of hierarchical regression models show that the models suggested are capable of predicting between 37 and 65% of the variance of the variables of interest. These models give support (albeit partial) to the hypotheses stated. Meanwhile, the analyses based on fsQCA indicated that none of the variables was a necessary condition to achieve high levels of credibility, loyalty, or identification and informed of the distinct combinations of factors that were sufficient to achieve high and low levels of the variables of interest.

The results of the hierarchical regression models confirm that service quality influences credibility, but not necessarily loyalty or identification, which lose weight with the inclusion of the emotional variables. Therefore, there seems to be partial support for the first hypothesis raised, which posited that *The service quality positively and significantly influences credibility, loyalty, and identification.* This, on the one hand, would be in line with the idea that credibility is highly associated with the capacity of the organization to create value and satisfy the expectations of its members (Oriade and Schofield, 2019; Theodorakis et al., 2019; Vuong et al., 2020), and achieving it requires control to be maintained over the correct implementation of activities and processes. Something similar happens with the influence of transformational and transactional leadership on the variables of interest (H2: *Transformational leadership and transactional leadership positively and significantly influence credibility, loyalty, and identification).* The results confirm the hypothesis 2 only for the case of transformational leadership on credibility, but not for loyalty or identification that loses significance with the inclusion of the emotional variables. Given the influence that transformational leadership has on credibility, this would be critical in defining the type of relationships that sportspeople and amateurs establish with their leaders, thus confirming that pointed out by Arnold et al. (2015) and Mitrovic et al. (2019). Particularly, the results obtained in the first step of the multiple regression analyses confirm that transformational leadership is a better precursor of loyalty than transactional leadership (Monzani et al., 2014), but unlike that study, these differences would be statistically significant.

In the analysis of the emotional variables, as in the cases before, the hypotheses associated with their influence on credibility, loyalty, and identification of amateur sportspeople [H3: Affective commitment and emotional attachment (investment and dividend) positively and significantly affect credibility, loyalty, and identification.

H4: The emotional variables of emotional attachment (investment and dividend) and affective commitment are better predictors of credibility, loyalty, and identification than the management variables (service quality, transformational leadership, and transactional leadership)] were only partially confirmed.

However, it was shown that the inclusion of these variables improved the predictive capacity of the models significantly in trying to explain loyalty and identification while not significantly improving the case of credibility. This offers additional support for the idea that affective commitment and attachment are central in defining the attitudes of individuals, especially in those factors that determine long-term relationships with people or institutions (Morgan and Hunt, 1994; Geyskens et al., 1996). On the other hand, it reinforces the argument of Zajonc and Markus (1982) and Park and MacInnis (2006) in relation to the value of emotional aspects over and above more cognitive and management-based elements (such as service quality) when the time comes to predict answers related to commitment (such as loyalty and identification).

In terms of the results of the qualitative comparative analysis, in addition to what was already stated about none of the variables being necessary, the sufficiency analyses for the prediction of high values of credibility are able to explain 47% of the cases. The most representative models by order of capacity for explanation are the interaction between high service quality, high levels of transformational leadership, and low levels of transactional leadership (30%). Other observed combinations were the interaction between high levels of service quality, low levels of transformational leadership, and high levels of transactional leadership (29%) and the interaction between high levels of transformational leadership, low levels of transactional leadership, and high levels of emotional attachment dividend (29%). In the same way as the results drawn out by the structural regression analysis, these results reaffirm the importance of the variables associated with the service quality to achieve high levels of credibility (in only one of the models does a high emotional variable appear). It also reaffirms the idea that the service quality is a precursor for credibility, as, according to Crespo-Hervás et al. (2019), from the service quality would depend on the value the sporting institution is able to create for its members, as well as its capacity to satisfy their expectations (Vuong et al., 2020). These results are also in line with the idea that a good service quality helps to build the trust of the members of an organization and thus credibility (Men, 2012).

In the prediction of high levels of loyalty, different sufficient conditions explain 86% of the cases. The most relevant combination includes high levels of transformational leadership and high levels of emotional attachment dividend (72%). Then, this is followed by the combination of high levels of service quality, high levels of emotional attachment investment, and high levels of emotional attachment dividend (67%). Finally, high levels of transactional leadership and high levels of emotional attachment dividend (67%) are also associated with high levels of loyalty. These results confirm the idea of Lee and Ding (2020) that both transformational leadership and transactional leadership can be useful in improving the commitment of people within an organization, one by means of the task and the other by means of inspiration. This also gives support to the conclusions of Peng et al. (2020) with respect to the idea that transformational leadership is a source of inspirational motivation. This, in combination with affective commitment, would improve the levels of the loyalty of people within a sporting organization. In the same way, the results discussed around the loyalty of amateur footballers in this study are in line with Alexandris et al. (2017), who point out that the quality of the service in the organization of sporting events is aligned with high levels of loyalty of the fans, only in this case, it is required for this variable to interact with emotional attachment investment and with emotional attachment dividend. Thus, the conclusions of Heere and Dickson (2008), who point out that loyalty depends on both internal (affective) factors and external (management) ones, are confirmed.

Finally, in the prediction of identification, it is observed that to obtain high levels of this variable, there are distinct paths that explain 88% of the cases. The most relevant combination is the interaction between high levels of transformational leadership and high levels of emotional attachment dividend (73%). Then, it is observed that high levels of affective commitment to the club and high levels of both emotional attachment investment and emotional attachment dividend (70%) are also aligned with high levels of identification with the club, as are high levels of transactional leadership and high levels of emotional attachment dividend (69%). These results, just as in the case of the models associated with loyalty, confirm the importance of the emotional variables with their presence in all the models that explain high levels of identification. These results reinforce the ideas stated by Preston and Brown (2004) and Hoye (2007) that suggest that affective commitment influences the performance of voluntary workers, and this would occur as a consequence of greater stability in their link to these institutions. Distinct to loyalty, identification incorporates a feeling of belonging to the organization (Boehm et al., 2015) that would explain in this case the presence of the emotional variables in all the models associated with high identification. The results also show the relevance of the two types of leadership studied in combination with the emotional variables, confirming what was stated by Miao et al. (2013) and Lee and Ding (2020) who found that leadership increases the trust in the supervision of the leader, and this, in turn, boosts the feeling of belonging.

This study also has certain limitations. The use of questionnaires is the first of them, although it is widely used, can give way to a certain bias of desirability. In addition, the size of the sample, together with the fact that the data only consider Chilean clubs, makes the extracted results very local and not easy to generalize. In spite of this, the results of the study suppose a contribution for managers of sporting entities, offering tools to understand the influence of emotions in the management of organizations and thus be able to obtain adequate levels of identification and loyalty toward them, which supposes a clear benefit in their success and sustainability.

### Contributions and Implications

Looking at the results, the QCA models of analysis seem to be more explicative than the linear models. On the other hand, the results suggest that professionals related with the management of sporting organizations, apart from concentrating on organizational variables, should also focus on emotional variables, such as commitment, emotional attachment dividend, and emotional attachment investment in order to obtain high levels of loyalty and identification.

On the other hand, it has been demonstrated that the use of both methodologies can be very interesting due to the fact that HRM contributes data related to the individual impact of the variables, while the QCA allows for what is known as equifinality, which consists of the possibility that there can be different interactions that can arrive by different paths to the same result (Woodside, 2013; Rey-Martí et al., 2016; Giménez-Espert and Prado-Gascó, 2018).

The results obtained provide tools to understand the influence of emotions in the management of organizations, enabling managers of sporting entities to promote the success and sustainability of their organizations.

## Data Availability Statement

All datasets presented in this study are included in the article/Supplementary Material.

## Ethics Statement

The studies involving human participants were reviewed and approved by Scientific Ethics Committee of the Universidad Católica de la Santísima Concepción, Chile. Written informed consent for participation was not required for this study in accordance with the national legislation and the institutional requirements.

## Author Contributions

All authors listed have made a substantial, direct and intellectual contribution to the work, and approved it for publication.

## Conflict of Interest

The authors declare that the research was conducted in the absence of any commercial or financial relationships that could be construed as a potential conflict of interest.
